# Ubiquitin-specific peptidase 48 regulates Mdm2 protein levels independent of its deubiquitinase activity

**DOI:** 10.1038/srep43180

**Published:** 2017-02-24

**Authors:** Kateřina Cetkovská, Hana Šustová, Stjepan Uldrijan

**Affiliations:** 1 Department of Biology, Faculty of Medicine, Masaryk University, Brno, Czech Republic; 2 International Clinical Research Center, St. Anne’s University Hospital, Brno, Czech Republic

## Abstract

The overexpression of Mdm2 has been linked to the loss of p53 tumour suppressor activity in several human cancers. Here, we present results suggesting that ubiquitin-specific peptidase 48 (USP48), a deubiquitinase that has been linked in previous reports to the NF-κB signaling pathway, is a novel Mdm2 binding partner that promotes Mdm2 stability and enhances Mdm2-mediated p53 ubiquitination and degradation. In contrast to other deubiquitinating enzymes (DUBs) that have been previously implicated in the regulation of Mdm2 protein stability, USP48 did not induce Mdm2 stabilization by significantly reducing Mdm2 ubiquitination levels. Moreover, two previously characterized USP48 mutants lacking deubiquitinase activity were also capable of efficiently stabilizing Mdm2, indicating that USP48 utilizes a non-canonical, deubiquitination-independent mechanism to promote Mdm2 oncoprotein stability. This study represents, to the best of our knowledge, the first report suggesting DUB-mediated target protein stabilization that is independent of its deubiquitinase activity. In addition, our results suggest that USP48 might represent a new mechanism of crosstalk between the NF-κB and p53 stress response pathways.

Tumour suppressor p53 modulates important cellular processes such as senescence, cell cycle arrest, apoptosis, and DNA repair in response to various stress stimuli, including DNA damage, hypoxia, ribosomal stress, telomere erosion, and oncogene activation. The activity of p53 is tightly controlled by several factors, including the E3 ubiquitin ligase Mdm2 and a related protein Mdm4 (MdmX), both of which seem to be critical for suppressing the antiproliferative activity of p53 in normal somatic cells and during embryonic development^1^,^2^,^3^. On the other hand, Mdm2 and MdmX have been found to be overexpressed in many human cancers, contributing to the loss of the tumour-suppressive function of p53 in cancer cells^4^.

Protein ubiquitination mediated by E3 ubiquitin ligases such as Mdm2 or the Mdm2/MdmX complex and the subsequent p53 protein degradation in 26S proteasomes are key regulatory events in the p53 pathway. Another level of regulation is provided by deubiquitinating enzymes (DUBs), which mediate the removal of the ubiquitin moiety, often leading to increased stability of their target proteins. The human genome encodes at least 98 DUBs that can be subdivided into six families based on their sequence and structural similarity, of which the ubiquitin-specific peptidases (USPs) with over 50 members constitute the largest DUB family^5^,^6^. While DUBs may be functionally as important as ubiquitin ligases, many of their roles in the regulation of cellular homeostasis are poorly understood.

USP7 (also known as HAUSP) was the first DUB found to be involved in the regulation of the p53 pathway, with HAUSP overexpression resulting in p53 stabilization^7^. However, depletion of HAUSP did not decrease cellular p53 levels, as predicted, but instead led to an increase in p53 levels. These studies suggest that the regulation of the p53 pathway by this DUB is a complex process in which Mdm2, rather than p53, is the main target of HAUSP^8^,^9^. Mdm2 seems to be the preferred substrate for USP7 in unstressed cells, and genotoxic stress decreases USP7 binding to Mdm2 through ATM-dependent phosphorylation, shifting the balance toward p53 stabilization^10^,^11^. While USP7 localizes mainly to cell nuclei with only a fraction of USP7 present in the cytoplasm^12^, USP10, a different DUB of the USP family, could be involved in the deubiquitination of cytoplasmic p53. Upon DNA damage, USP10 can translocate to the nucleus and also contribute to p53 activation^13^,^14^. USP42 has been identified as a p53-interacting DUB whose activity contributes to the fine-tuning of p53 activity in cells recovering from mild or transient damage^15^. USP24 is another DUB that was recently implicated in the regulation of the p53 pathway and in the cellular response to DNA damage by deubiquitinating p53^16^. In contrast to these deubiquitinases that target p53, USP2a was shown to deubiquitinate and stabilize only Mdm2 and Mdm4 while exhibiting no deubiquitinase activity toward p53^17^,^18^. The ectopic expression of USP2a leads to Mdm2 and Mdm4 stabilization and promotes p53 degradation, and USP2a knockdown increases cellular p53 protein levels and transcriptional activity. In addition to these DUBs that directly target the main players in the p53 pathway, several USPs have been shown to modulate the p53 pathway activity by targeting other p53 regulators, rather than Mdm2. For instance, USP4 was shown to reduce p53 levels via deubiquitination of ARF-BP1^19^. Interestingly, some USPs might be preferentially required for p53 activation in response to a certain stress stimulus. For example, USP28 cooperates with 53BP1 to activate p53 in response to centrosome loss and prolonged mitosis but has no effect on doxorubicin-induced p53 accumulation^20^,^21^.

Ubiquitin-specific peptidase 48 (USP48, also known as USP31) was shown to bind TRAF2, a RING finger E3 ubiquitin ligase and an important activator of NF-κB signaling, and was reported to be able to cleave lysine 63- and lysine 48-linked polyubiquitin chains *in vitro*^22^,^23^. USP48 overexpression can lead to inhibition of TRAF2-mediated NF-κB activation, possibly through USP48-mediated TRAF2 deubiquitination. A recent study showed that USP48 associates with the COP9 signalosome complex and promotes the nuclear accumulation of NF-κB RelA through its DUB activity, trimming K48-linked ubiquitin chains^24^. Given that NF-κB signaling can have a strong impact on the p53 pathway by controlling the cellular levels of Mdm2 ubiquitin ligase^25^,^26^,^27^, we sought to analyze the possible role of USP48 in the regulation of the p53 pathway. To our surprise, our results suggest that USP48 is also a new binding partner for Mdm2 and plays a direct role in the regulation of Mdm2 stability via a non-canonical, deubiquitinase activity-independent mechanism.

## Materials and Methods

### Cell culture

Human cell lines H1299, U2OS (ECACC, Salisbury, UK) and HEK293T (DSMZ, Braunschweig, Germany) were cultivated at 37 °C and 5% CO_2_ in a high-humidity atmosphere in Dulbecco’s modified Eagle’s medium (DMEM) supplemented with 10% fetal bovine serum, 2 mM glutamine, 100 U/ml penicillin and 100 μg/ml streptomycin sulfate (all from Sigma-Aldrich).

### Plasmid constructs

Plasmids coding for Flag-tagged wild-type USP48 (pcDNA3-Flag-USP48) and Flag-tagged USP48 mutants C98S and S1 were kindly provided by George Mosialos (Aristotle University of Thessaloniki, Greece). Mammalian expression plasmids coding for Flag-tagged wild-type p53 (in pcDNA3), HA-tagged wild-type ubiquitin (pMT123), wild-type human Mdm2 (pCHDM1A) and its derivatives (Mdm2 ΔAD (Δ245–95), Mdm2 ΔRING (Δ441–91), Mdm2 Δ212–347, Mdm2 Δ296–346, Mdm2 Δ347–417, Mdm2 Δ239–491, Mdm2 Δ 239–491 Δ58–89), and GFP-tagged Mdm2 constructs (pEGFP-C1: GFP-Mdm2 231–440, GFP-Mdm2 265–440, GFP-Mdm2 300–440) were kindly provided by Karen Vousden and Henning Horn (Beatson Institute for Cancer Research, Glasgow, UK). Plasmids coding for HA-tagged wild-type ubiquitin (pRK5-HA-Ubiquitin-WT) and ubiquitin mutants K48 and K48R were gifts from Ted Dawson (Addgene plasmids #17604, 17605 and 17608). Plasmids coding for enhanced GFP protein (pEGFP-N1 and pEGFP-C1) were obtained from Clontech. In all transfections, the total amount of DNA was kept constant using the empty plasmid construct pcDNA3 (Invitrogen).

### Knockdown of USP48 expression

Mixtures of small interfering RNAs (siRNAs) targeting the human *USP48* transcript and non-targeting control siRNAs were purchased from Santa Cruz Biotechnology. Hiperfect transfection reagent (QIAGEN) was used for siRNA transfection into the U2OS and H1299 cell lines according to the manufacturer’s protocol. Cells were collected 48 h post-transfection, washed with PBS, lysed in 2x SDS sample buffer, and analyzed by SDS-PAGE and Western blotting. An anti-Mdm2 mouse monoclonal antibody (Ab-1) (Merck Millipore) and an anti-USP48 rabbit polyclonal antibody (Abcam) were used to detect the endogenous levels of Mdm2 and USP48, respectively.

### Analysis of protein-protein interactions by immunoprecipitation

Cells were transfected in 100-mm plates with plasmids coding for wild-type Mdm2 or Mdm2 deletion mutants and wild-type or mutant Flag-USP48 (C98S and S1) in a total amount of 6 μg of DNA using Lipofectamine 2000 reagent (Thermo Fisher Scientific). The cells were treated with proteasome inhibitor MG132 (15 μM in DMEM; Sigma-Aldrich) 24 h post-transfection. Four hours later, the cells were washed with ice-cold PBS and lysed in an IP lysis buffer (150 mM NaCl, 50 mM Tris-HCl pH 8.0, 1% Triton X-100) containing protease inhibitors (Complete Mini EDTA-free; Roche Diagnostics) by incubating on ice for 30 min, and the extracts were centrifuged (13.000 rpm, 30 min, 4 °C). Immunoprecipitations were performed at 4 °C with 1 μg of antibody (anti-Mdm2 Ab1 mouse monoclonal antibody (Merck-Millipore), anti-GFP mouse monoclonal antibody (Roche), or anti-Flag mouse monoclonal antibody M2 (Sigma-Aldrich)). After incubating with the antibody overnight, 20 μl of protein G-Sepharose beads (GE Healthcare) were added to the lysate and incubated for 45 min on a rotating wheel at 4 °C. The beads were washed three times with the IP lysis buffer and resuspended in 2x SDS sample buffer. Proteins from the whole-cell extracts and immunoprecipitations were resolved by SDS-PAGE and analyzed by Western blotting.

### Immunofluorescence

U2OS cells grown on glass coverslips in 60-mm dishes were transfected with plasmids coding for wild-type Flag-USP48 using Lipofectamine 2000 reagent (Invitrogen). Twenty-four hours post-transfection, the cells were washed with PBS and fixed in 3% paraformaldehyde for 20 min at room temperature. After fixation, the cells were washed with PBS, permeabilized with 0.2% Triton X-100 in PBS (5 min) and blocked with PBS containing 0.5% bovine serum albumin (30 min, room temperature). Coverslips were incubated with primary antibodies recognizing endogenous Mdm2 (Ab-1, mouse monoclonal; Merck-Millipore) and the Flag tag in USP48 (anti-Flag rabbit polyclonal antibody; Sigma Aldrich) for 2 h in the blocking solution. After washing three times with PBS, the coverslips were incubated for 1 h with a mixture of DyLight^TM^ 594 conjugated anti-mouse and fluorescein isothiocyanate (FITC)-conjugated anti-rabbit antibodies (Jackson ImmunoResearch Laboratories) in the blocking solution containing 1 μg/ml DAPI (Sigma-Aldrich) for visualization of cell nuclei. The coverslips were washed with PBS and mounted on microscopic slides using Vectashield mounting medium (Vector Laboratories). Images were taken using a FluoView^TM^ 500 confocal laser scanning fluorescence microscope (Olympus).

### Protein stability profiling – cycloheximide chase assay

For protein stability profiling, 20 μM cycloheximide (Sigma-Aldrich) was added to the growth medium 10, 30 or 60 min prior to cell harvesting. Treated and control cells were washed with cold PBS and lysed in 2x SDS sample buffer, and the proteins were resolved by SDS-PAGE and detected by Western blotting. PCNA was detected as a loading control (PC-10 antibody; kindly provided by Borek Vojtesek, Masaryk Memorial Cancer Institute, Brno, Czech Republic). Mdm2 protein levels were analyzed using ImageJ software (http://rsbweb.nih.gov/ij/) and expressed in graphs relative to PCNA protein levels.

### Mdm2 and p53 protein degradation assays

U2OS cells in 60-mm dishes were transfected with a total of 3 μg of plasmid DNA using Lipofectamine 2000 reagent (Invitrogen). For analyses of p53 degradation, an equal amount of plasmid construct coding for Flag-tagged p53 was co-transfected with an empty plasmid, a wild-type Mdm2 plasmid construct, or the Mdm2 plasmid together with the Flag-USP48 expression construct. For analyses of Mdm2 proteasomal degradation, Mdm2 was expressed either alone or together with increasing amounts of Flag-USP48. Each transfection mixture contained 0.5 μg of pEGFP-N1 to control for the transfection efficiency. The total amount of DNA in the individual transfection mixtures was kept constant by adding the empty plasmid pcDNA3 construct. After transfections, the cells were cultured for 24 h, washed with PBS and lysed with 2x SDS sample buffer. Proteins were resolved by SDS-PAGE and analyzed by Western blotting. Anti-Mdm2 (Ab-1, mouse monoclonal; Merck-Millipore) and anti-Flag (M2, mouse monoclonal; Sigma Aldrich) antibodies were used to detect ectopically expressed Mdm2 and USP48 proteins, respectively.

### Mdm2 and p53 ubiquitination assays

To detect the ubiquitination levels of Mdm2 or p53, U2OS cells grown in 60-mm dishes were transfected with a total amount of 6 μg of plasmid DNA using Lipofectamine 2000 reagent (Invitrogen). Each transfection mixture contained 0.5 μg of HA-ubiquitin wild-type construct (or HA-tagged mutant ubiquitin K48 or K48R). The cells were treated 24 h post-transfection with 15 μM MG132 (Sigma-Aldrich) for 3 h and lysed in 300 μl 0.5% SDS. The lysates were boiled for 5 min, vortexed, cooled to room temperature and diluted with 1 ml of Triton X-100 lysis buffer (see paragraph Immunoprecipitations). The immunoprecipitation of Mdm2 was performed using 1 μg of anti-Mdm2 Ab1 antibody. For p53 immunoprecipitation, 1 μg of DO-1 mouse monoclonal antibody was used (kindly provided by Borek Vojtesek, Masaryk Memorial Cancer Institute, Brno, Czech Republic). After a two-hour incubation on a rotating wheel, 20 μl of protein G-Sepharose (Sigma-Aldrich) were added for another 45 min. The beads were washed three times with Triton X-100 lysis buffer and boiled in 2x sample buffer, and the released proteins were resolved by SDS-PAGE and analyzed by Western blotting using anti-HA mouse monoclonal antibody (F-7; Santa Cruz Biotechnology) to detect ubiquitin levels.

## Results

### USP48 contributes to the regulation of Mdm2 expression

**Figure 1:**
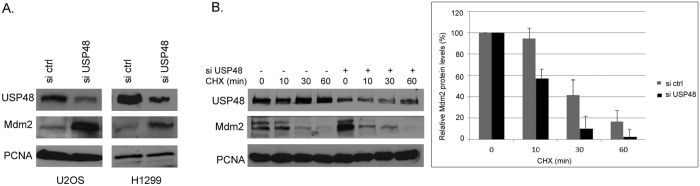
Endogenous USP48 contributes to regulation of Mdm2 expression in cancer cells. (**A**) Two human cancer cell lines (U2OS and H1299) were transfected with a mixture of control non-targeting siRNAs (si ctrl) or siRNAs targeting USP48 expression (siUSP48). Levels of endogenous Mdm2 and USP48 were analyzed using SDS-PAGE and Western blotting. Endogenous PCNA served as a loading control. (**B**) U2OS cells transfected with control or USP48 siRNAs were treated with cycloheximide (CHX) and samples collected at various time points were subjected to SDS-PAGE and Western blotting to analyze changes in the levels of USP48 and Mdm2 (left). The graph, representing data obtained in three independent experiments (means + standard deviation), shows relative changes in the protein levels of Mdm2 at different time points after the addition of CHX in control or USP48 siRNA-transfected cells.

To determine whether USP48, through its ability to regulate NF-κB transcription factor, could play a role in the regulation of Mdm2 expression, we induced an siRNA-mediated knockdown of *USP48* in human osteosarcoma U2OS cells and human lung carcinoma H1299 cells. We observed significantly enhanced Mdm2 expression in the cancer cells with down-regulated USP48 levels (Fig. 1A), suggesting that USP48 could contribute to the regulation of Mdm2 expression in human cells. As H1299 cells do not express *p53*, this result also indicated that the effect of USP48 knockdown on Mdm2 expression was p53-independent. However, the analysis of protein turnover using the translation inhibitor cycloheximide (CHX) revealed that Mdm2 protein stabilized by the siRNAs targeting *USP48* was less stable than Mdm2 in the cells treated with the control non-targeting siRNAs (Fig. 1B), suggesting an additional role of USP48 as a regulator of Mdm2 protein stability. To further characterize this unexpected role of USP48, we performed a series of experiments with ectopically overexpressed Mdm2 and USP48 proteins.

### USP48 promotes Mdm2 stability

**Figure 2:**
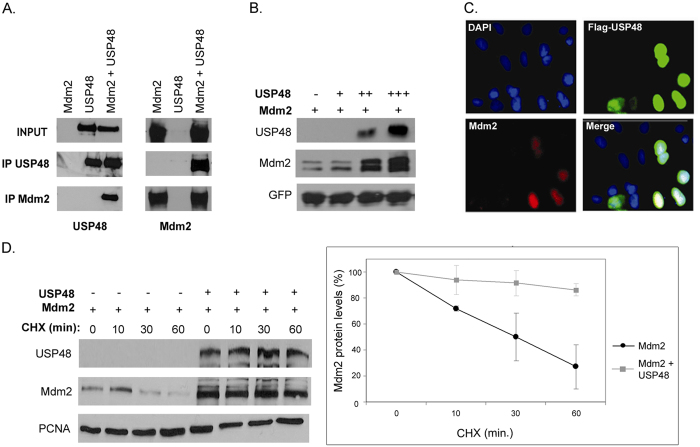
USP48 interacts with Mdm2 and promotes its stability. (**A**) Mdm2 interacts with USP48 in immunoprecipitations. Flag-tagged USP48 and Mdm2 were overexpressed in HEK293T and immunoprecipitations were performed using anti-Mdm2 or anti-Flag antibodies. Immunoprecipitates were analyzed by SDS-PAGE and Western blotting. (**B**) USP48 co-expression causes dose-dependent increase in Mdm2 protein levels. Mdm2 was overexpressed together with increasing amounts of Flag-USP48 in U2OS cells. GFP plasmid was used as a transfection control. Cells were lysed 24 h post-transfection, lysates resolved by SDS-PAGE and individual proteins detected by Western blotting. (**C**) USP48 transfection induces increase in endogenous Mdm2 protein levels. U2OS cells grown on coverslips were transfected with Flag-USP48, fixed 24 h later, and immunofluorescence was used to determine Mdm2 and USP48 levels in individual cells. DAPI was used for visualizing cell nuclei. (**D**) USP48 promotes Mdm2 protein stability. U2OS cells were transfected with Mdm2 and Flag-USP48 plasmids. After 24 h, cells were treated with cycloheximide (CHX) and harvested at different time points and Mdm2 protein levels were analyzed by Western blotting using Ab-1 antibody. PCNA served as a loading control. Mdm2 and PCNA protein levels were analyzed using ImageJ software and relative changes in Mdm2 levels were calculated. Data presented in the graph represent results of three independent experiments (means+/− standard deviation).

First, we performed co-immunoprecipitations of overexpressed Mdm2 and FLAG-tagged USP48, and the results indicated that the two proteins might be able to form complexes in cells (Fig. 2A). More detailed subsequent analyses of Mdm2-USP48 interactions suggested the presence of two independent USP48-binding sites in Mdm2 (Supplementary data, Figure S1). The first USP48 binding site appeared to be located within the acidic domain (amino acids 260–300), in a region required for p53 ubiquitination and degradation and involved in Mdm2 binding to several known Mdm2 regulators. The second site is probably located at the N-terminus of Mdm2, but it does not seem to comprise the region required for p53 binding (amino acids 58–89).

To test the possible effect of USP48-Mdm2 interactions on Mdm2 protein stability, Mdm2 was expressed in U2OS cells together with increasing amounts of USP48. We found that the co-expression of USP48 can lead to a dose-dependent increase in Mdm2 protein levels (Fig. 2B). Using immunofluorescence microscopy, we detected increased levels of endogenous Mdm2 protein in U2OS cells transiently transfected to ectopically express high levels of USP48 (Fig. 2C).

To determine whether the observed USP48-mediated Mdm2 upregulation might reflect increased stability on the protein level, we performed a cycloheximide (CHX) chase assay in U2OS cells transfected either with the Mdm2 construct alone or in combination with USP48. Cycloheximide was added 24 h post-transfection to inhibit protein synthesis, and samples were collected at different time points after CHX treatment. While in the absence of USP48 overexpression, the Mdm2 protein exhibited rapid turnover with a half-life of approximately 30 minutes, and the stability of Mdm2 protein was significantly increased in the presence of ectopically expressed USP48 (Fig. 2D).

### USP48 can enhance Mdm2-mediated p53 ubiquitination and degradation

**Figure 3:**
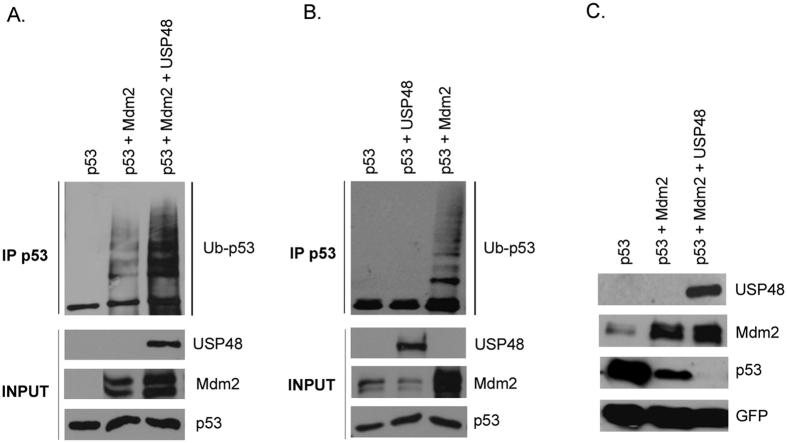
USP48-mediated Mdm2 stabilization leads to increased p53 ubiquitination and degradation. U2OS cells were transfected with Flag-p53, Mdm2, Flag-USP48 and hemagglutitin (HA)-tagged ubiquitin. Cells were treated 24 h post-transfection with 15 μM MG132 for 3 h and lysed in 0.5% SDS. Lysates were boiled for 5 min, vortexed and diluted with Triton X-100 lysis buffer. Immunoprecipitation of p53 was performed using anti-p53 monoclonal antibody DO-1 for 2 h. Immunoprecipitates were analyzed by SDS-PAGE and Western blotting (**A,B**). U2OS cells were transfected with Flag-p53, Mdm2, Flag-USP48 and GFP encoding plasmids. Cells were lysed 24 h post-transfection in 2x SDS sample buffer, proteins resolved by SDS-PAGE and analyzed by Western blotting (**C**).

As Mdm2 is best known as a critical negative regulator of p53, in the next set of experiments, we analyzed the impact of the observed USP48-mediated Mdm2 stabilization on cellular p53 protein levels and p53 ubiquitination. While the co-expression of Mdm2 and USP48 strongly enhanced p53 ubiquitination (Fig. 3A), USP48 was not capable of inducing p53 ubiquitination in the absence of Mdm2 (Fig. 3B), suggesting that the observed increase in Mdm2 levels was largely responsible for the enhancement of p53 ubiquitination in the presence of USP48. Importantly, p53 degradation assay showed that the USP48-induced increase in Mdm2-mediated p53 ubiquitination could also lead to more efficient p53 degradation (Fig. 3C).

### Mdm2 stabilization does not require USP48 deubiquitination activity

**Figure 4:**
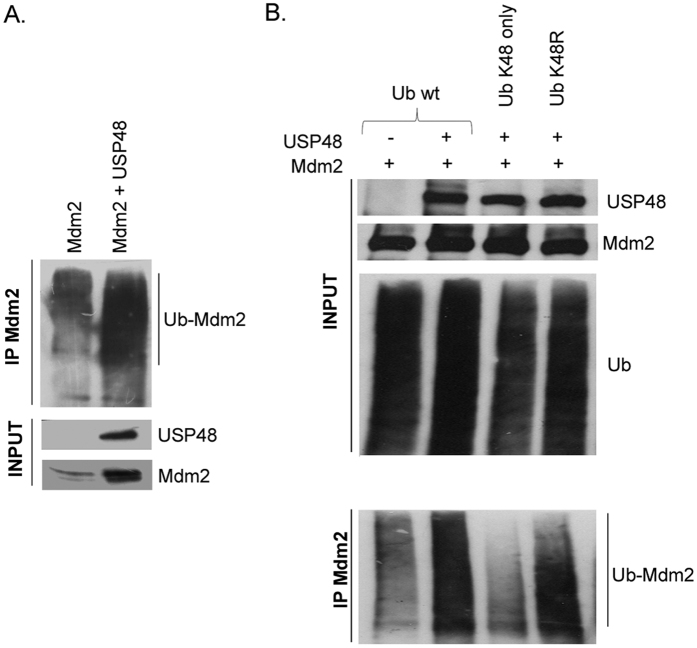
Mdm2 is not deubiquitinated in the presence of USP48. U2OS cells were transfected with Mdm2, Flag-USP48, and HA-tagged wild type ubiquitin plasmid construct pMT123 (**A**) or pRK5-derived HA-ubiquitin constructs: wild-type, K48, and K48R (**B**). Ubiquitination assay was performed as described in Fig. 3, but instead of p53, Mdm2 was immunoprecipitated from the lysate using Ab-1 antibody and Mdm2 ubiquitination was detected using anti-HA antibody.

**Figure 5:**
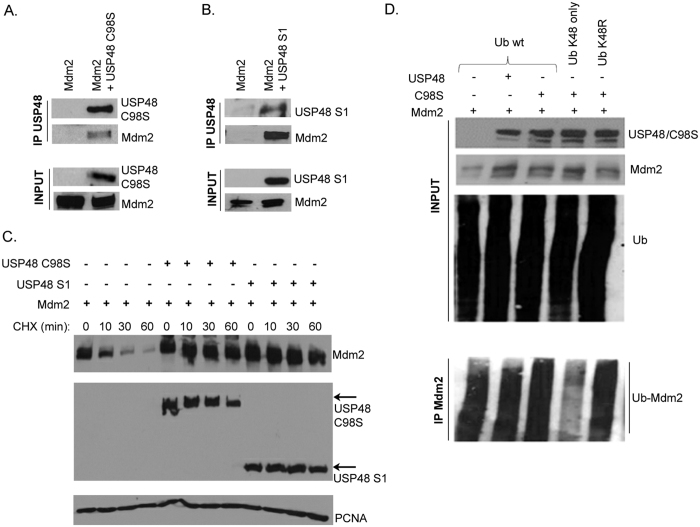
USP48 deubiquitinase activity is not required for Mdm2 stabilization. Mdm2 interacts with deubiquitinatinase-defective USP48 mutants C98S (**A**) and USP48 S1 (**B**). Immunoprecipitations were performed as described in Fig. 2A. (**C**) Deubiquitination-defective USP48 mutants are capable of stabilizing Mdm2. U2OS cells were transfected with Mdm2, Flag-USP48 C98S or Flag-USP48 S1 expression plasmids. After 24 h, cycloheximide chase assay was performed as described in Fig. 2D. (**D**) U2OS cells were transfected with Mdm2, Flag-USP48 mutant C98S, and pRK5-derived HA-ubiquitin constructs: wild-type (wt), K48, and K48R. Ubiquitination assay was performed as described in Fig. 4.

DUBs can stabilize target proteins by removing covalently attached ubiquitin moieties or ubiquitin chains, and Mdm2 deubiquitination has been proposed to be responsible for Mdm2 stabilization in the presence of USP7 and USP2a^11^,^17^. We therefore tested the possibility that Mdm2 might also be a substrate for the previously reported deubiquitinating activity of USP48. We were surprised to find that USP48 caused Mdm2 stabilization that was not accompanied by a significant decrease in the overall levels of Mdm2 ubiquitination, which was unexpected (Fig. 4A). On the other hand, USP48 has been reported to trim rather than completely disassemble K48-linked Ub chains^24^, and the presence of other types of ubiquitin linkages to Mdm2 might be capable of masking the effects of USP48 on K48-linked ubiquitin. To exclude this possibility, in our ubiquitination assays we not only used the plasmid construct coding for wild-type ubiquitin (Ub wt) but also two ubiquitin mutants: one allowing for Ub attachment only through lysine 48 (Ub K48only) with all remaining lysine residues in the ubiquitin molecule mutated to arginine, and the second ubiquitin with lysine 48 mutated to arginine but maintaining all the other lysine residues intact and capable of mediating the linkage of ubiquitin (Ub K48R). We observed that fewer ubiquitin molecules were attached to Mdm2 via the K48 residue of Ub than through other lysines in the presence of USP48, which could be the reason for the less efficient proteasomal degradation of Mdm2 when USP48 was co-expressed (Fig. 4B). However, if the active trimming of K48-linked ubiquitin was responsible for Mdm2 stabilization, USP48 protein lacking deubiquitinase activity should not be able to modulate Mdm2 protein levels. To test this, we employed two USP48 mutants previously reported to be deubiquitination defective. While the first mutant (USP48 C98S) contained a single cysteine to serine exchange at position 98 within the catalytic domain, the second mutant tested was a short isoform of USP48 (USP48-S1) missing the C-terminal region of the protein that was shown to lack deubiquitinating activity and the expression of which was found to be upregulated in several types of cancer cells compared to normal tissues^23^,^24^. First, we confirmed that both deubiquitination-defective mutants were still able to physically interact with Mdm2 in co-immunoprecipitations (Fig. 5A,B). Surprisingly, the cycloheximide chase experiments then showed that both USP48 mutants were also capable of promoting the stability of Mdm2 protein in cells (Fig. 5C). These results clearly showed that the stabilization of Mdm2 by USP48 did not depend on its deubiquitinase activity. Interestingly, when we analyzed the pattern of Mdm2 ubiquitination in the presence of the deubiquitinase-defective mutant USP48 C98S, we again observed that less ubiquitin was attached to Mdm2 via lysine 48 compared to other lysines, suggesting that the deubiquitinase activity of USP48 was not responsible for the previously observed difference and supporting our notion that USP48 did not target Mdm2 with its deubiquitinase activity.

## Discussion

Many DUBs can act as regulators of protein stability owing to their ability to remove ubiquitin chains that serve as signals for proteasomal degradation^5^. Some of these enzymes have been shown to participate in the regulation of the p53 tumour suppressor pathway by modulating the ubiquitination of p53 or its major negative regulator Mdm2^10^,^11^,^13^,^17^,^20^,^21^.

In this study, we analyzed a possible role of USP48 in the regulation of the p53 pathway. USP48 is a poorly characterized protein, and few reports regarding its function and regulation are available. The first published functional analysis of USP48 activity suggested that USP48 possesses deubiquitinase activity and that it might exhibit higher activity toward lysine 63-linked ubiquitin compared to lysine 48-linked ubiquitin, at least *in vitro*^23^. However, a later report showed that USP48 is also active toward K48-linked ubiquitin, trimming the K48-linked Ub chains rather than completely disassembling them^24^. Both reports implicated deubiquitinase activity of USP48 in the regulation of NF-κB signaling. In our experiments, USP48 was identified as a new interacting partner for the ubiquitin ligase Mdm2. Its ability to promote Mdm2 stability suggested that it might behave in a manner similar to other previously identified DUBs that are capable of deubiquitinating Mdm2, such as USP7/HAUSP or USP2a^11^,^17^, thereby preventing the proteasomal degradation of Mdm2. However, our analysis of the overall levels of Mdm2 ubiquitination did not reveal a significant decrease. This result was further supported by our unexpected finding that two USP48 mutants lacking deubiquitinase activity were also capable of stabilizing cellular Mdm2 protein levels, and these data collectively speak against the requirement of Mdm2 deubiquitination for its stabilization by USP48. Moreover, these results also suggested that USP48 does not mediate Mdm2 stabilization through the deubiquitination of other Mdm2 regulators. Interestingly, we found that only a relatively small fraction of ubiquitin molecules is linked to Mdm2 via K48 in the presence of USP48, but this does not seem to reflect its deubiquitinase activity, as the same pattern of K48 ubiquitin linkage to Mdm2 was also observed in the presence of deubiquitinase-inactive USP48 mutants. Nevertheless, we cannot exclude the possibility that USP48 (and its deubiquitinase inactive mutants) are capable of recruiting another deubiquitinase responsible for trimming K48-linked ubiquitin, and future studies are necessary to determine the exact mechanism by which USP48 promotes Mdm2 stability. One possible explanation could be provided by our finding that USP48 binds to the central acidic domain of Mdm2, a region that was shown by us and others to independently contribute to p53 ubiquitination and to p53 and Mdm2 stability^28^,^29^,^30^,^31^,^32^. The acidic domain was also reported to mediate Mdm2 interactions with the regulatory subunits of the 26S proteasome and seems to play a role in Mdm2 homodimerization^33^,^34^, both of which might contribute to the regulation of Mdm2 degradation as well as to Mdm2 stabilization if disrupted by the binding of USP48 to the acidic domain.

Crosstalk between the p53 and NF-κB transcription factors can play a pivotal role in determining cellular responses to stress^25^,^26^,^35^. The Mdm2 protein itself has been reported to bind and inhibit the p65Rel subunit of NF-κB^36^. As already mentioned, deubiquitinases have been shown to play regulatory role in the NF-κB pathway – not only USP48 but also A20, CYLD, Cezanne, and USP21^23^,^24^,^37^. Our data suggest that, in addition to serving as an NF-κB regulator, through the direct regulation of Mdm2 protein stability, USP48 might represent a novel mechanism of crosstalk between the p53 and NF-κB pathways.

Taken together, our data indicate that USP48 deubiquitinase could serve as a new modulator of the p53 pathway with a non-canonical mechanism of action, potentially contributing to the up-regulation of Mdm2 levels in cancer cells. To the best of our knowledge, this is the first example of DUB-mediated protein stabilization not requiring deubiquitination of the target protein.

## Additional Information

**How to cite this article**: Cetkovská, K. *et al*. Ubiquitin-specific peptidase 48 regulates Mdm2 protein levels independent of its deubiquitinase activity. *Sci. Rep.* **7**, 43180; doi: 10.1038/srep43180 (2017).

**Publisher's note:** Springer Nature remains neutral with regard to jurisdictional claims in published maps and institutional affiliations.

## Supplementary Material

Supplementary Figure S1
