# Supplementary material for: Ubiquitin-specific peptidase 48 regulates Mdm2 protein levels independent of its deubiquitinase activity
Source: Sci Rep. 2017 Feb 24;7:43180. doi: 10.1038/srep43180 (PMC5324091; doi:10.1038/srep43180)
Supplement: Supplementary Figure S1 [file srep43180-s1.pdf]

Supplementary Information

Ubiquitin-specific peptidase 48 regulates Mdm2 protein levels independent of its deubiquitinase activity

Kateřina Cetkovská[a, b], Hana Šustová[a], Stjepan Uldrijan[a, b,*]

[a]Department of Biology, Faculty of Medicine, Masaryk University, Brno, Czech Republic
[b]International Clinical Research Center, St. Anne's University Hospital, Brno, Czech Republic
[*] Corresponding author:
E-mail: uldrijan@med.muni.cz

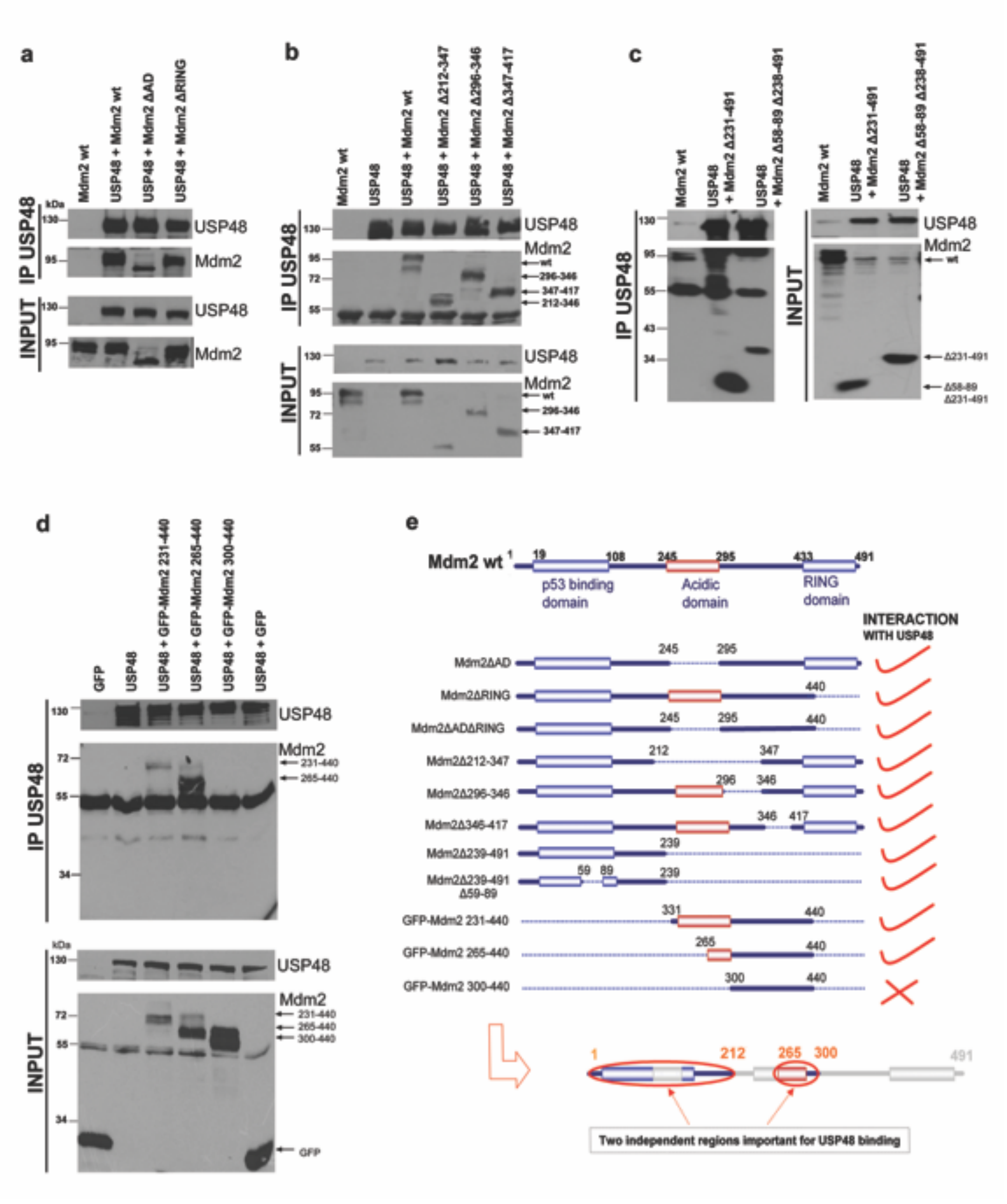

Figure S1

Mapping of USP48 binding to Mdm2. HEK293T cells were transfected with an expression plasmid coding for Flag-tagged USP48 in combination with plasmids encoding wild type Mdm2 or Mdm2 deletions mutants (**a-c**) or GFP-tagged central domain of Mdm2 (**d**). Cells were lysed 24h post-transfection and USP48 was immunoprecipitated using anti-Flag M2 antibody (Sigma-Aldrich). Immunoprecipitates were resolved by SDS-PAGE and analyzed by Western blotting, using anti-Mdm2 Ab-1 (Merck Millipore), anti-Flag M2, and anti-GFP (Roche) antibodies. (**e**) Schematic representation of individual Mdm2 constructs used in the immunoprecipitations and their interaction with USP48.
